# Integrating patient-reported physical, mental, and social impacts to classify long COVID experiences

**DOI:** 10.1038/s41598-023-43615-8

**Published:** 2023-09-28

**Authors:** Keri Vartanian, Daniel Fish, Natalie Kenton, Benjamin Gronowski, Bill Wright, Ari Robicsek

**Affiliations:** 1 Center for Outcomes Research and Education (CORE), Providence St. Joseph Health, 5251 NE Glisan Street, Portland, OR USA; 2 Providence Research Network, 1801 Lind Ave SW, Renton, WA USA

**Keywords:** Viral infection, Health services, Quality of life

## Abstract

Long COVID was originally identified through patient-reported experiences of prolonged symptoms. Many studies have begun to describe long COVID; however, this work typically focuses on medical records, instead of patient experiences, and lacks a comprehensive view of physical, mental, and social impacts. As part of our larger My COVID Diary (MCD) study, we captured patient experiences using a prospective and longitudinal patient-reported outcomes survey (PROMIS-10) and free-text narrative submissions. From this study population, we selected individuals who were still engaged in the MCD study and reporting poor health (PROMIS-10 scores < 3) at 6 months (n = 634). We used their PROMIS-10 and narrative data to describe and classify their long COVID experiences. Using Latent Class Analysis of the PROMIS-10 data, we identified four classifications of long COVID experiences: a few lingering issues (n = 107), significant physical symptoms (n = 113), ongoing mental and cognitive struggles (n = 235), and numerous compounding challenges (n = 179); each classification included a mix of physical, mental, and social health struggles with varying levels of impairment. The classifications were reinforced and further explained by patient narratives. These results provide a new understanding of the varying ways that long COVID presents to help identify and care for patients.

## Introduction

Early clinical reports of COVID-19 described the course of clinical infection to be 2–6 weeks depending on severity. Patients, however, were telling a different story: as early as March 2020, COVID-19 patients began to share their stories of post-acute sequalae of symptoms and experiences lasting well beyond initial infection^1^. These patients began to call themselves “long-haulers,” and by May 2020, the term “long COVID” had gained widespread adoption. This patient-led movement changed our collective understanding of the disease and carried significant implications for individual patients and society at large.

Long COVID has been described as a syndrome encompassing extended physical and neuropsychiatric symptoms that persist longer than 12 weeks^2,3^, although descriptions and definitions vary. Several studies examining long COVID have documented symptoms at six months and even a year post-acute infection^4–10^. Estimated prevalence of long COVID varies across studies^11^—likely in part because of varied study populations, definitions, and follow-up time – with estimates generally ranging from 10 to 30% of patients^11,12^. Clinical and patient-reported manifestation of long COVID include varied and diverse symptoms such as dyspnea, metabolic disorders, cardiovascular disorders, gastrointestinal disorders, musculoskeletal pain, malaise, fatigue, headaches, neurocognitive disorders, loss of taste/smell, memory loss, concentration issues, and mental health disorders^2,5,8,9,13–15^.

In addition to physical and mental health symptoms, the COVID-19 pandemic has had profound social impacts, including on family dynamics, personal relationships, work-life balance, work environments, economic security, and other aspects of daily living^16^. These social factors are widely known to be connected to our physical and mental health^17,18^. However, our knowledge of the “social experiences” of long COVID and how they connect with patients’ physical and mental health remains limited. A multidimensional view of the patient experience of long COVID, including its physical, mental, and social manifestations, can help healthcare systems provide appropriate long-term care to support patient recovery.

Long COVID experiences vary widely, but researchers have made progress attempting to develop general long COVID clusters or typologies^2,14,19,20^. These studies have primarily focused on physical symptoms: Huang et al. for example, leveraged electronic health records to find five physical symptom clusters 60 days after initial infection^19^. A separate large study out of the United Kingdom used surveys and free-text responses to identify two clusters of physical symptoms after 12 weeks^14^. While these studies begin to shed light on potential physical health classifications of long COVID, they only partially describe the long COVID experience because they lack inclusion of mental and emotional health as well as the social impacts of COVID-19. This expansion is critical as we know physical, mental, and social health are inextricably linked and must be considered together to appropriately manage and treat the large population of patients with long COVID.

In this study, we leverage data from My COVID Diary (MCD), a prospective, longitudinal study conducted by a multi-state health system in the Western United States, to identify and understand clusters of long COVID symptoms and experiences from the patient perspective. MCD utilizes a standard patient-reported outcomes scale in combination with open-ended narrative journal entries, collected longitudinally to document patients’ COVID-19 experiences over time. We use this data to describe patient-reported outcomes, perform latent class analysis (LCA) to create integrated long COVID classifications, and to understand patient narratives of their long COVID experiences. Our approach is built on two key principles: that an understanding of long COVID should be multidimensional and include its physical, mental, and social impact domains, and that it should be firmly rooted in the voices of the patients who have experienced it.

## Results

### Sample characteristics

Demographic characteristics of the study sample are shown in Table 1. The study sample included a total of 634 participants, of which 68% were Female and 58% were White, with a mean age of 51 years (SD = 14.4). Over half (52%) reported having been hospitalized or visited the Emergency Department (ED) for COVID-19 at some time since their reported infection. Demographic characteristics of our sample differed from the general MCD participant population in the following ways: participants in the study sample were more likely to be Female, more likely to be older (Age 50–64), and more likely to have visited the ED or been hospitalized for COVID-19.

**Table 1:** Demographics.

Characteristic	Gen. MCD population^1^, N = 10,664^2^	Study sample, N = 634^2^
Sex		
Female	6269 (59%)	428 (68%)
Male	3403 (32%)	148 (23%)
Other/Unknown	992 (9.3%)	58 (9.1%)
Race		
White	6147 (58%)	369 (58%)
Hispanic/Latino	1915 (18%)	92 (15%)
Multiracial	466 (4.4%)	44 (6.9%)
Black	326 (3.1%)	27 (4.3%)
Asian	403 (3.8%)	16 (2.5%)
Other/Unknown	1407 (13%)	86 (14%)
Age		
18–29	1639 (15%)	43 (6.8%)
30–49	4364 (41%)	230 (36%)
50–64	2987 (28%)	248 (39%)
65 +	1673 (16%)	113 (18%)
ED^3^	3545 (33%)	327 (52%)
Weeks since onset^4^, avg. (SD)	33.0 (24.9)	53.3 (16.2)
Global T scores^5^ at 20–28 weeks		
Physical Health, avg. (SD)	N/A	39.8 (7.8)
Mental Health, avg. (SD)	N/A	41.1 (14.4)

^1^Study sample not included.

^2^n (%).

^3^Ever visited ED or hospitalized for COVID-19.

^4^Number of weeks since first positive test or onset of symptoms at the time of the study. Each patient’s onset date was used as their initial time point for analysis.

^5^Calculated from PROMIS-10 scores at 20–28 weeks.

The average Patient-Reported Outcomes Measurement Information System (PROMIS-10) Physical and Mental Global T scores for our study sample were 39.8 (SD = 7.8) and 41.1 (SD = 7.3), respectively, a full standard deviation or nearly a full standard deviation below the population norm of 50, indicating a cohort with meaningfully lower functional health than the general population. Initial PROMIS-10 scores 1–4 weeks post-infection for this same cohort were 42.3 for their Physical Global T score (SD = 8.7), and 43.5 for their Mental Global T score (SD = 8.4), indicating that rather than improving over time, our cohort of long COVID participants experienced modest declines in their global health over the five to six months since their initial weeks of illness. Additional details on the distribution of Global Health T scores in our study sample are available in the Supplementary Information (Supplementary Figs. 1 and 2). Distributions of scores for individual component questions from the PROMIS-10 can also be found in the Supplementary Information (Supplementary Fig. 3).

### LCA PROMIS-10 analysis and narrative data

#### LCA model results

**Figure 1:**
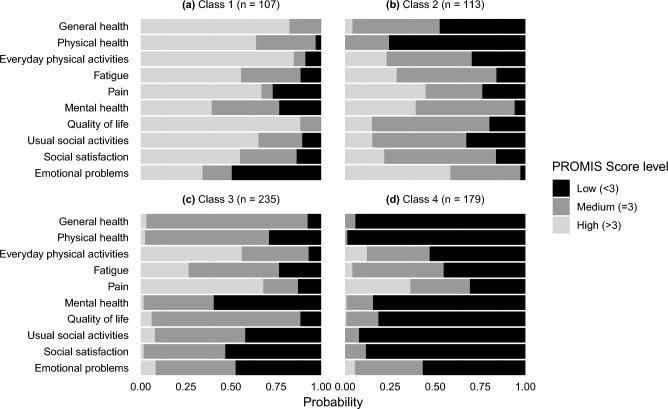
Conditional class membership probabilities for PROMIS-10 scores at 6 months. In each panel, the length of the shaded bars represents the probability for a particular response being given membership in a certain class. For example, the probability of reporting a ‘Low’ Emotional Problems score for Class 1 was 50%, which is indicated by the black horizontal bar spanning 50% of the Emotional Problems row in the Class 1 panel, while the probability of reporting a ‘High’ General Health score for Class 2 was 4.1%, which is indicated by the light shaded horizontal bar spanning only 4% of the General Health row in the Class 2 panel.

Evaluation and comparison of model fit parameters for PROMIS-10 scores suggested that a Latent Class Analysis (LCA) model with four classes was an optimal fit to the data (see Supplementary Fig. 4 and Supplementary Table 2), and each participant was assigned to one of the four LCA classes based on model probabilities. The number of participants assigned to each LCA class ranged from 107 to 235. Demographics across all four classes were similar, with those in Class 2 and 4 being slightly older and more likely to have been hospitalized for COVID-19 (Supplementary Table 1). Classification diagnostics indicated a high degree of accuracy for class assignment. Conditional class probabilities along with class sizes after assignment are depicted in Fig. 1. Detailed class probability tables and model fit data (including AIC, BIC, Chi-squared, and G-square) are outlined in the Supplementary Information (Supplementary Fig. 4 and Supplementary Tables 2, 3 and 4).

#### Narrative data

Individuals in every class discussed a variety of physical and mental health symptoms alongside social and daily impacts of COVID-19. These experiences were often connected as individuals described multiple challenges. Within these narratives, certain themes emerged that described and illuminated the types of experiences that characterize membership in each class of long COVID identified in the LCA model.

#### Overview of results

The PROMIS-10 scores and themes from the narrative analysis were combined and reviewed to create a profile of predominant features for each identified class. We ultimately identified four classifications or “groupings” of long COVID experiences:

- *Class 1. A Few Lingering Issues (17% of sample):* These participants generally reported good functional health across all domains, but they were experiencing a few lingering problems that kept them from feeling like they were completely recovered.
- *Class 2. Significant Physical Symptoms (18% of sample)*: These participants reported multiple lingering physical symptoms that were significant enough to impede their overall functioning and day-to-day activities 5–6 months after infection.
- *Class 3. Ongoing Mental & Cognitive Struggles (36% of sample):* These participants reported fewer physical symptoms but multiple mental health and cognitive challenges that were significant enough to impact their social lives and day-to-day functioning 5–6 months after infection.
- *Class 4. Numerous Compounding Challenges (28% of sample):* These participants reported managing multiple physical, mental, and cognitive challenges, which significantly impacted their overall quality of life.

A summary of these classifications is provided in Table 2. PROMIS-10 data and narrative themes for each class are provided below.

**Table 2:** Summary of Long COVID Experiences at 20–24 Weeks.

Class	Class name	n (% of sample)	Low domains per person*	Analysis summary	Example quote
1	A few lingering issues	107 (17%)	1.5 (0.9)	Generally doing better but still have a few lingering symptoms they are managing; they are typically able to maintain a good quality of life. The challenges of the pandemic environment are negatively impacting them and causing emotional challenges and social isolation	*“Sometimes feel fatigued, tired. I am able to perform all daily tasks and work effectively.”*
2	Significant physical symptoms	113 (18%)	2.8 (1.5)	Managing multiple physical symptoms, especially physical fatigue. Their physical health is negatively impacting their mental health. They struggle with their daily activities, work, and social interaction	*“I still continue to cough and have shortness of breath, hair loss, fatigue, blurred vision and headaches on and off.”*
3	Ongoing mental & cognitive struggles	235 (36%)	2.9 (1.5)	Mental health and cognitive decline are predominant, and they also are managing physical symptoms. Their challenges, especially brain fog and cognitive issues, make it hard to complete daily activities and work. Social interaction is difficult due to these struggles and because these interactions can cause more anxiety	*“Emotional challenges is an understatement, but yes the anxiety makes me irritable or more vulnerable. Personal life and work life are being greatly affected by the anxiety itself.”*
4	Numerous compounding challenges	179 (28%)	7.3 (1.5)	Managing multiple physical, mental, and cognitive challenges. Their numerous symptoms are negatively impacting their daily activities and ability to work. Social interaction is difficult and overwhelming	*“Sat/Sun cried all day, as very hard to function w/ brain fog, extreme fatigue, no taste or smell, swollen joints.”*

*Average Number of Low PROMIS-10 domains ( >) per person, avg (SD) out of 10 domains measured.

#### Class 1: a few lingering issues

Class 1 contained 17% of participants (n = 107) and was characterized by generally favorable PROMIS-10 scores in all components. Participants in this class were likely to report favorable outcomes (greater than 50% chance of a response of either ‘High’ or ‘Medium’) in all components except for Mental Health and Emotional Problems. The most likely ‘High’ outcome in Class 1 was Quality of Life (88.3%) and the most likely ‘Low' outcome was Emotional Problems (49.6%). For Class 1, the average number of low PROMIS-10 domains per person was ~ 1.5 (distribution in Supplementary Fig. 5). Overall, PROMIS-10 scores told a story of participants who were doing relatively well in terms of functional health, but still had some lingering symptoms or challenges.

Participant narratives confirmed this picture. By 5–6 months post infection, individuals in Class 1 reported that they were feeling better and that life was generally good, but a mix of lingering physical and mental health challenges prevented them from feeling fully “recovered.” There were reports of frustration and emotional challenges, often associated with restrictions due to the pandemic (such as lockdown, working from home, limitations on social interaction), which were associated with negative impacts on social satisfaction and left some people feelings isolated. In general, people reported that they were able to continue their daily activities despite still not feeling quite like themselves. Key quotes include:

*“I‘m feeling better overall”.*

*“The social constraints of lockdown continue to be challenging. Just overall anxiety all the time”.*

*“[My] irritability like relates to excessive workload caused by pandemic”*

*“My physical health is fine, but I don’t feel so great mentally”*

*“Life is lonely. Lonelier. Many activities I used to do are no longer available”.*

*“Sometimes feel fatigued, tired. I am able to perform all daily tasks and work effectively”.*

#### Class 2: significant physical symptoms

Class 2 represented 18% of the study sample (n = 113). These participants were characterized by significant physical health issues reported through their PROMIS-10 scores that were severe enough to impact their functional quality of life. Probability of poor outcomes (‘Low’ scores) for Class 2 was higher for questions about Physical Health (75.7%) and General Health (47.6%) and lower for Emotional Problems (2.8%), Mental Health (6.0%), and Social Satisfaction (16.4%). For Class 2, the average number of low PROMIS-10 domains per person was ~ 2.8 (distribution in Supplementary Fig. 5). Overall, PROMIS-10 scores for this group told a story of participants with impaired ability to live their usual lives attributable to lingering physical symptoms of COVID-19.

Journal entries for these participants reinforced this picture. Participants in Class 2 journaled about their physical symptoms, especially fatigue, that even after 5–6 months was severe enough to be debilitating and impact their normal lives. Some were experiencing mental health challenges, but this was often explicitly associated with frustration about their lingering physical symptoms. Brain fog and cognitive issues were infrequent in this group, and when present, they tended to be improving. Support from family and friends seemed to help them push through their challenges and maintain social connection. However, physical health challenges were impacting their daily lives including their work, daily activities, and socializing. Some people were able to start exercising, but it tended to be more difficult than expected. Key quotes include:

*“Still struggling with back pain, getting PT, but it is frustrating and depressing”*

*“I still continue to cough and have shortness of breath, hair loss, fatigue, blurred vision and headaches on and off”.*

*“I do not have enough energy to work and participate in social activities like I did before Covid. I get down because of these limitations”.*

*“The brain fog seems to be getting better, l have been able to finish a few things and figure out how to fix a couple of new projects”.*

*“The fatigue I can push through mostly even though I don't want to, but having a family is the biggest incentive to get up and keep going”.*

*“I have begun exercising but only manage one bike ride a week right now”.*

#### Class 3: ongoing mental and cognitive struggles

Class 3 represented the largest proportion of participants (36%, n = 235) and their PROMIS-10 scores were characterized by ongoing mental health issues, with relatively high probability of poor outcomes (‘Low’ scores) for questions about Mental Health (59.6%), Social Satisfaction (53.3%), and Emotional Problems (47.5%), and relatively better scores in the physical health domains (Everyday Physical Activities and General Health). For Class 3, the average number of low PROMIS-10 domains per person was ~ 2.9 (distribution in Supplementary Fig. 5). PROMIS-10 scores in this group told a story of participants whose physical symptoms had largely subsided but who were still struggling with cognitive or mental health challenges such as brain fog, lack of focus, or depression that significantly impeded their quality of daily living.

These experiences were supported by participant journal entries, as individuals in Class 3 wrote about a range of mental health challenges, including reports of anxiety, depression, and low motivation. Some linked their mental health challenges to continued physical symptoms, but many reported mental health as a major challenge on its own. Cognitive impacts, including brain fog, memory loss, poor thinking ability, and lack of focus, were prevalent and sometimes worsening over time. Engaging in social activities would frequently cause stress and anxiety due to fear of infection or because of their continued struggles (especially their mental/emotional challenges). Some participants reported that they avoided engaging in social activities because of their continued mental health challenges, compounding feelings of loneliness and isolation. Their challenges, especially mental and cognitive, negatively impacted their ability to work and perform their usual activities. Individuals in Class 3 also reported returning to their exercise routines, but some still found exercise a struggle. Key quotes include:

*“Emotional challenges is an understatement, but yes the anxiety makes me irritable or more vulnerable. Personal life and work life are being greatly affected by the anxiety itself”.*

*“Mental issues are the biggest problem”.*

*“I’ve been experiencing anxiety and depression since being so sick from Covid realizing that my lungs will never be the same is such a harsh reality!!”*

*“Brain fog and trouble concentrating are still making school and work difficult, as is the constant fatigue”.*

*“Cognitive skills are decreasing. Even had a fire in the kitchen from lack of simple attention. Forgetfulness increasing”.*

*“Running as an exercise is still difficult. I have to constantly stop as opposed to being able to continuously run”.*

#### Class 4: numerous compounding challenges

Class 4 contained 28% of participants (n = 179) and was characterized by relatively poor outcomes (greater than 60% chance of ‘Low’ or ‘Medium’ response) in all PROMIS-10 components and more than a 50% chance of a ‘Low’ response in all components except for Pain and Fatigue. For Class 4, the average number of low PROMIS-10 domains per person was ~ 7.3 (distribution in Supplementary Fig. 5). PROMIS-10 scores for this group indicated that these participants were struggling in almost every dimension of health that the PROMIS-10 scale is designed to measure.

Individuals in Class 4 wrote in their journal entries about their many and varied continuing symptoms. Most individuals in this class reported having multiple symptoms and challenges they were managing. They had persistent physical fatigue and physical health challenges. Their mental health challenges, including anxiety and depression, often stemmed from their poor physical health, social isolation, and the pandemic environment. Many were experiencing brain fog and cognitive issues impacting memory, thinking, and focus. Their persistent symptoms were having major negative impacts on their ability to perform basic daily functions (such as walking upstairs), complete daily tasks (such as work and chores), and engage in social activities. When participating in social activities, they became tired, overwhelmed, and anxious. Key quotes include:

*“Muscle weakness, joint pain, constant headache, neck stiffness and pain, racing heart, muscle spasms, hands and fingers freeze. In the afternoon I can’t organize my brain, loss of words heavy fatigue, brain fog”.*

*“Anxiety with any social situation including grocery store or any store…severe panic attacks and stress”*

*“Sat/Sun cried all day, as very hard to function w/ brain fog, extreme fatigue, no taste or smell, swollen joints”.*

*“Brain fog, loss of comprehension, sentence structure difficulties and cannot remember common words when attempting to speak”.*

*“I am always exhausted. I am trying to work… [but] I haven’t been able to work a whole week since I came back from Covid. I have no energy to go or do anything”.*

*“I have stairs I have to climb at my apartment and I go up the stairs I can’t hardly breathe”.*

To confirm that patterns of responses in the four classes observed at 5–6 months were not an artifact of the PROMIS-10 domains, we did a separate LCA analysis using PROMIS-10 data from the first 4 weeks of illness. Results showed a different pattern of responses across the four classes without a significant distinction between physical and mental health outcomes (Supplementary Fig. 6).

## Discussion

This study centers the patient experience through MCD, which prospectively tracks longitudinal data from patients who test positive for COVID-19 across the health system’s seven state footprint. The goal of this study was to leverage this rich data to understand long COVID experiences by integrating patient-reported outcomes and patient narratives across physical, mental, and social impacts at six months. Using LCA, four distinct classes of long COVID emerged, and qualitative analysis of narrative data reinforced and further described the patient experiences of these four classes. The results create a working model of four types of long COVID patient experiences: 1. A Few Lingering Issues; 2. Significant Physical Symptoms; 3. Ongoing Mental & Cognitive Struggles, and 4. Numerous Compounding Challenges. All four types of experiences had physical, emotional, and social experiences and impacts, but our analysis reveals some of the differences in the predominant types of experiences that patients are managing six months after infection. While a limited number of studies have examined patient-reported physical, mental/emotional, and social functioning for patients experiencing long COVID^21,22^, to our knowledge, this is the first study to integrate validated survey responses and patient narratives to characterize and understand types of long COVID experiences.

Other studies that have performed LCA analysis on COVID-19 symptoms and experiences have focused either on the physical symptoms of acute COVID-19, such as studying clinical phenotypes using LCA^23–25^, on broad mental health outcomes such as subgroup analysis of self-reported mental health^26^, or LCA analysis of the response to social distancing measures and associated mental health measures^27^. Our study combines physical, mental, and social patient-reported outcomes with rich patient narratives to deeply explore the different kinds of long COVID experiences. We found that the narrative context reinforced and further detailed the patterns of experiences reported in the PROMIS-10. To our knowledge, no other LCA analysis of COVID-19 has leveraged this comprehensive view of patient outcomes and narratives to understand long COVID experiences. It is interesting to note that the four types of experiences we describe show similarities to other LCA analyses of physical and psychological symptoms of cancer patients^28^, suggesting that the types of experiences related to managing long COVID may relate to other chronic diseases.

There is currently no known treatment for long COVID, but some guidelines have been developed for primary care^29^, and hospital systems have begun to launch long COVID care programs. These guidelines and programs aim to treat the specific needs of the individual patient by assembling care teams across physical health specialties to manage the myriad of long COVID symptoms. Our data demonstrates the critical importance of taking an integrated, “whole person” approach to managing long COVID, with multidisciplinary teams that include significant mental health and social supports. Across all four classes of long COVID, participants reported that they are managing a combination of physical, mental, and social impacts that intersect with one another in complex ways. Interestingly, “Ongoing Mental & Cognitive Struggles” was our largest classification, and all three other classifications had some struggles with mental health—emphasizing the importance of mental health care in long COVID. Further, the unique health challenges and impacts that stem from living and being sick during a pandemic, such as social isolation, must be considered and addressed as deliberately as lingering physical and cognitive symptoms. Long COVID is an intersectional health challenge that impacts the whole person; our responses to long COVID must be the same.

This study has several key limitations. The study sample lacks racial diversity and potentially other unmeasured levels of diversity such as gender identity or socioeconomic status. MCD is only offered in English and therefore we also lack experiences of patients with preferred languages other than English. People included in this study were seen at a hospital or clinic within the health system for a COVID-19 test and therefore may represent a sicker population than those who tested in other settings such as at home. Participants in the study needed to engage in a mobile platform to consent and for data collection, which may have excluded individuals with limited technology access or literacy. To be eligible for inclusion in our study sample, participants were required to continue their engagement in the research study for at least six months, which may have created bias in our sample and limit generalizability by excluding individuals no longer journaling at this time, possibly due to symptom resolution or poor health that limited their ability to participate. At this time, we are unable to connect to medical records to confirm any reported clinical manifestations; however, it is also unclear whether some of the types of experiences reported through MCD would be captured in an electronic medical record. Understanding how patient-reported outcomes and clinical records align is an important topic for future research. Additionally, while collapsing PROMIS-10 scores to a three-point scale facilitated interpretation, it may have led to decreased measurement sensitivity. Furthermore, this study did not capture health prior to COVID-19 infection, so we are unable to account for co-morbidities or baseline patient-reported health. Participants included in the study sample were infected with COVID-19 no later than September 2021, which was prior to the emergence of the Omicron variant and therefore may not represent long COVID experiences for newer variants and subvariants.

Patient reporting and activism identified long COVID as a clinical phenomenon. This study leverages longitudinal structured and unstructured patient-reported experiences across physical, mental, and social impacts to characterize four types of long COVID. It is from this interconnected dynamic of physical, mental, and social challenges that the full long COVID experience emerges, and, once again, the patient perspective has broadened our view and understanding of this disease. This characterization helps expand clinicians understanding of how to recognize and diagnose long COVID in their patients, as well as reveals the complex interaction between physical, mental, and social health that must be addressed to support patient recovery.

## Methods

### Study design

This study uses prospective longitudinal data collected through MCD to perform an observational cross-sectional analysis of participants who were still experiencing health issues 6 months after the onset of COVID-19 infection. Participation in MCD is offered across the health system’s seven-state footprint (Alaska, Oregon, Washington, California, Montana, New Mexico, and Texas). Since August 2020, any patient 18 years or older with a positive polymerase chain reaction (PCR) test at any location within the health system receives a text message within several days of their test inviting them to participate. Multiple contact attempts are made before exiting them from the protocol. If a patient consents to participate, they enroll via Twistle, a SMS-based HIPAA compliant patient engagement communication system, using e-consent and are asked to participate for up to one year. Enrollment is ongoing; to date (as of 10/8/2022), a total of 18,462 participants have consented to participate in MCD, representing 12% of total invitees and 27% of invitees who viewed the invitation message. Full study protocol was approved by Providence St. Joseph Health Institutional Review Board (IRB# 2020000467). All study procedures adhered to the required guidelines. For recruitment purposes only, we obtained a HIPAA Waiver of Consent to obtain contact information for patients with a new COVID-19 diagnosis. Patients were then contacted for study recruitment and informed consent was received through the Twistle app for all participants.

### Data collection

MCD participants receive regular data collection prompts via Twistle (every two days for the first two weeks, weekly from week 3 to 13, and then once a month for the rest of the year). At each timepoint, Twistle sends notifications to participants to collect both structured, validated questionnaires (e.g., the PROMIS-10 survey) and prompts for participants to write open-ended journal entries describing their symptoms and experiences. Participants submit their responses to the survey questions and free-text journal entries through Twistle and the data is then cleaned, ingested, and stored in a secure server for analysis.

#### Structured questionnaires

Global health outcomes are measured longitudinally via the PROMIS-10 (Patient-Reported Outcomes Measurement Information System) survey, a validated 10-item survey designed to assess physical, mental, and social health and well-being that has been used in other studies to help assess persistent post-acute COVID-19 symptoms (PROMIS-10 questions included in Supplementary Methods 1)^30–33^. Each PROMIS-10 component item is rated on a 1–5 scale with higher scores indicating better health, and two summary measures—Global Physical Health and Global Mental Health—are computed by summing the scores of the 10 components and transformed to standardized distributions with a population mean of 50 and standard deviation of 10.

#### Open-ended journal entries

Participant narratives are collected as open-ended journal entries in response the following prompts: (1) *“what were your first symptoms?”* and (2) *“please journal, in as many or as few words as you'd like, the problems you've been experiencing over the last few days. It may help to think about your body from head to toe. a. What is new? b. What has gotten worse? c. What has gotten better? d. What hasn't changed? e. Are you having emotional challenges? How is your personal and work life?”* After the first day, participants receive only the second prompt. Participants are free to respond as they like; no instructions are provided in terms of what information or level of detail to provide, and they can choose to stop journaling at any time or when their symptoms resolve. Entries are tracked and connected longitudinally to create a narrative record of each participant’s experiences and symptoms over time.

### Sample definition

**Figure 2:**
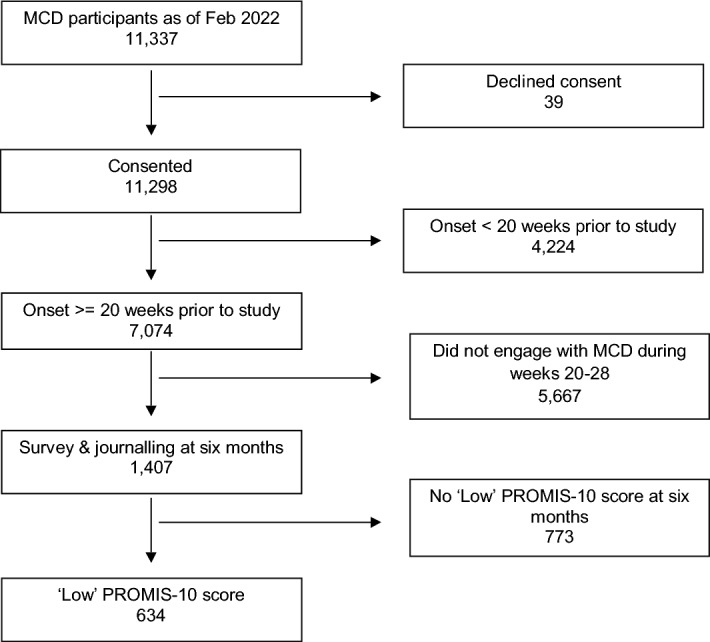
Study flow diagram for the inclusion or exclusion of MCD participants in the study.

For this analysis, we sought to identify and assess data for a subset of MCD participants who were still struggling with the effects of COVID-19 six months after their initial infection. To achieve this, we selected participants based on two key criteria: (1) participants who submitted at least one journal entry between 20 and 28 weeks after initial symptom onset, indicating at least some continuing COVID-19 symptoms five to six months after entering the study; and (2) participants who reported a low score (< 3) for at least one PROMIS-10 component question during that same time, indicating that participants were experiencing poor health in at least one dimension. Low PROMIS-10 component scores (< 3) are considered to be associated with poor health and higher scores (> 3) with good health^32–34^. The 20–28-week window was selected due to the monthly cadence of data collection at that point in MCD, which allowed us to include participants who may have missed one round of data collection. If a participant submitted PROMIS-10 responses more than once in the 20–28-week window, we selected submissions closest to each participant’s six-month mark (week 24). If a participant had more than one PROMIS-10 submission on a given day, one submission was selected at random. All journal submissions by a single participant on a given day were combined into a single submission. Our final sample was extracted on 2/9/2022 and contained 11,337 MCD participants. A total of 7074 individuals were 20 weeks or more past the date of their COVID-19 qualifying infection at the time of the study, 1407 of which were responding to the PRO surveys and providing journal entries within the 20–28-week window. Of these, a total of 634 individuals had low PROMIS-10 scores. These 634 MCD participants made up our sample for this study (Fig. 2).

### Quantitative analysis

Sample demographics were described with counts and proportions of this sample compared to the full MCD population. Descriptive analysis of PROMS-10 scores included statistical summary histograms of both Global and component Physical and Mental Health scores. We used Latent Class Analysis (LCA), a type of structural equation model that is used to find groups of responses in multivariate categorical data based on a probabilistic statistical model, to explore potential clustering and intersectionality of PROMIS-10 component scores and help identify “typologies” of long COVID experiences^28,35^. PROMIS-10 component scores were recoded as ordinal variables for ease of interpretation with three levels: ‘Low’ = 1–2, indicating least favorable outcomes, ‘Med’ = 3, and ‘High’ = 4–5, indicating most favorable outcomes. Since the study sample included only participants with at least one PROMIS-10 score less than 3 in the study window, every participant had a ‘Low’ score in at least one PROMIS-10 component^32,33^. The optimal number of classes to include in the LCA model was determined by evaluating and comparing model fit parameters for models of varying class size, including Bayesian information criterion, or BIC, Akaike information criterion, or AIC, Pearson’s Chi-square goodness of fit, and likelihood ratio chi-square^36–38^. After final model selection and parameterization, each participant was assigned a unique latent class using predicted posterior probabilities^36^. Classification accuracy was evaluated using relative entropy and average posterior class probability classification diagnostics^39^. No covariates were included in the model. All statistical analysis was performed using R Statistical Software^40^ and RStudio^41^. LCA analysis was performed using the poLCA package in R^36^.

### Qualitative analysis

To provide a more nuanced understanding of the experience of being in any given class, individual participant’s journal entries at 20–28 weeks were separated into four groups based on their assigned latent class and analyzed using Atlas.ti version 22. A thematic analysis approach was used to code and analyze the journal entries^42,43^. The codebook was developed deductively and inductively and organized into families that aligned with the PROMIS-10 categories; deductive development leveraged an existing codebook created for another portion of the MCD study, and inductive codebook development incorporated emergent codes as entries were coded. Previously coded journal entries were recoded as emergent codes were added. To ensure reliability of coding, one LCA group’s journal entries was randomly selected and 25% was double coded. Quotations associated with each code family were extracted and organized using a framework analysis approach^44^. The framework approach was used to break down the meaning behind the coded statements and to identify themes that described the experiences for the different LCA groups. Researchers coding and analyzing the qualitative data were blinded to specific group assignment. Final themes were reviewed by the research team and verified by checking them back against the data, including reviewing original documents and examining quantitative patterns of code frequencies.

### Supplementary Information

Supplementary Information.

## Data Availability

The datasets generated and/or analyzed during the current study are not publicly available due to identifying participant personal and health information, but de-identified survey data may be available from author Daniel Fish on reasonable request. Additionally, data collection is still ongoing. In the future, de-identified portions of the dataset may be made publicly available.
