# Supplementary material for: Integrating patient-reported physical, mental, and social impacts to classify long COVID experiences
Source: Sci Rep. 2023 Sep 28;13:16288. doi: 10.1038/s41598-023-43615-8 (PMC10539528; doi:10.1038/s41598-023-43615-8)
Supplement: Supplementary file 1 — Supplementary Information. [file 41598_2023_43615_MOESM1_ESM.pdf]

Title:

Integrating Patient-Reported Physical, Mental, and Social Impacts to Classify Long COVID Experiences

Authors & Affiliations:

Keri Vartanian, PhD[1]

Daniel Fish, PhD[1]

Natalie Kenton, MS, MPH[1]

*Benjamin Gronowski, MHR[1]

Bill Wright, PhD[2]

Ari Robicsek, MD[2]

1. Center for Outcomes Research & Education (CORE), Providence St. Joseph Health

   5251 NE Glisan Street, Portland, Oregon, USA

2. Providence Research Network

   1801 Lind Ave SW, Renton, Washington, USA

SUPPLEMENTARY INFORMATION

Supplementary Figure 1.

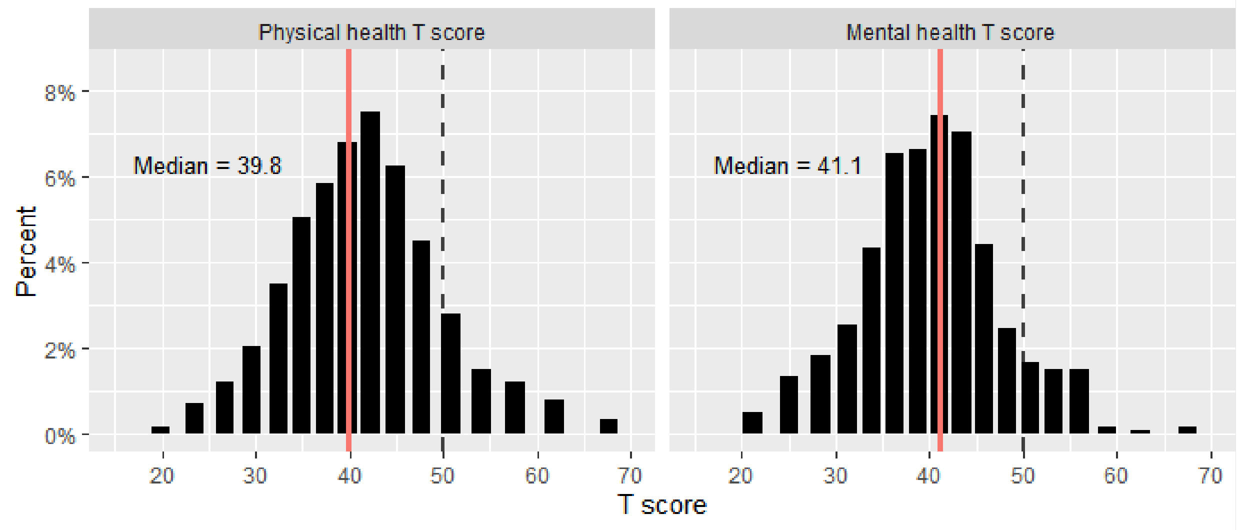

Supplementary Figure 1. Percent distributions of patient-reported Physical and Mental Health Global T scores at weeks 20-28 (N=634). Median scores are indicated by red vertical lines. Dotted lined represent the reference T score determined by the general US population mean.

Supplementary Figure 2.

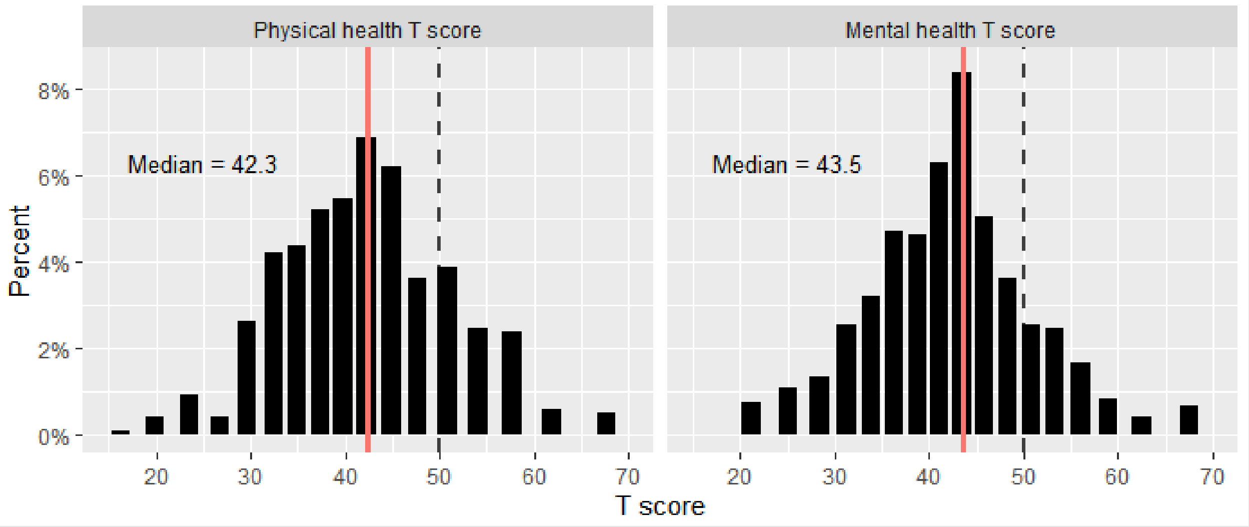

Supplementary Figure 2. Percent distributions of patient-reported Physical and Mental Health Global T scores at weeks 1-4 (N=597). Median scores are indicated by red vertical lines. Dotted lined represent the reference T score determined by the general US population mean.

Supplementary Figure 3.

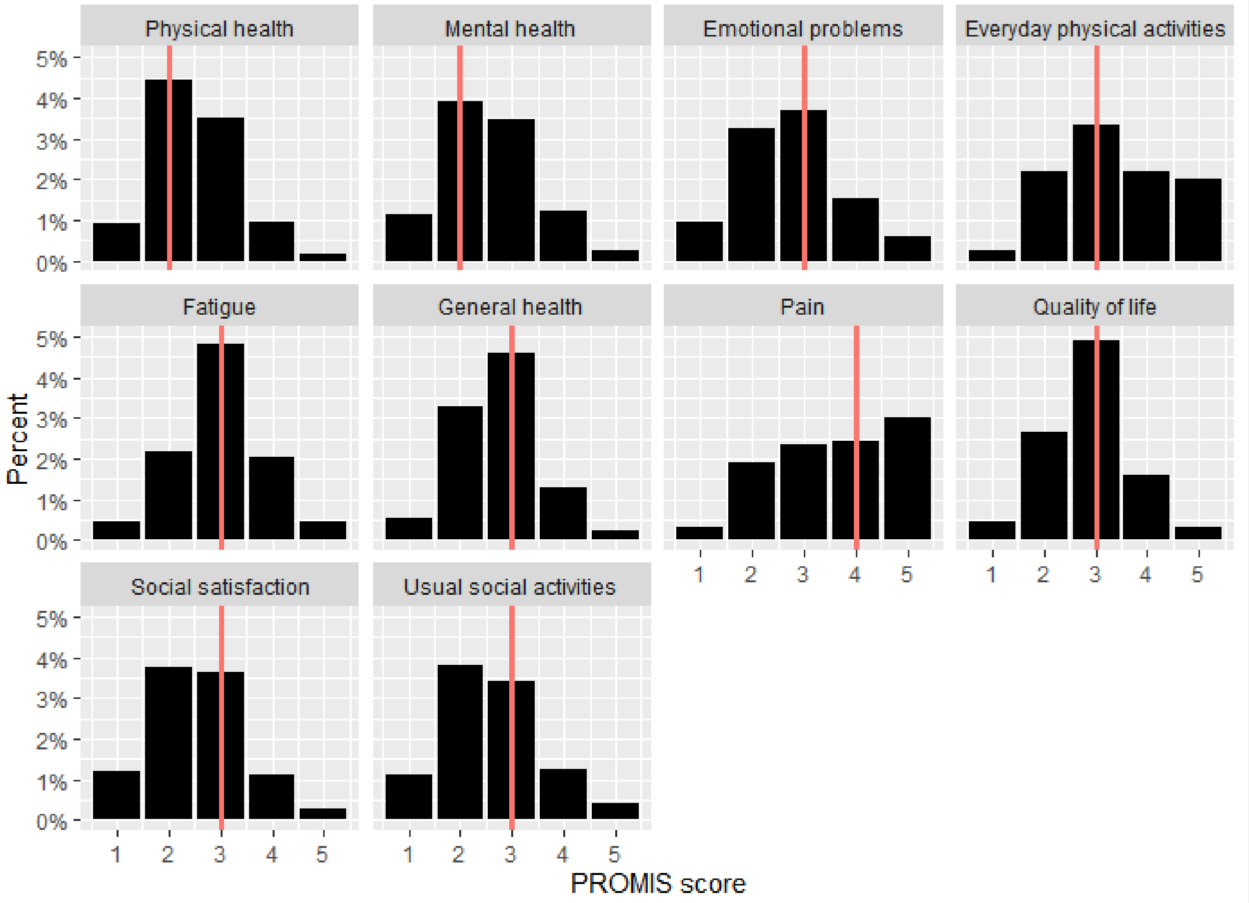

Supplementary Figure 3. Percent distributions of component scores reported by participants between weeks 20 and 28 for each PROMIS-10 component. Median scores are indicated by red vertical lines. Although inclusion criteria for the study required a score less than 3 for only one PROMIS-10 component, median scores did not exceed 3 for any of the components except Pain. In general, component distributions were centered around the median and distributions for most components were either symmetric or displayed slight right-skewness (i.e., scores clustered near the lower end of the scale) except for Pain and Everyday Physical Activities.

Supplementary Table 1. Demographic characteristics by LCA Class assignment.

Characteristic	Class 1<br>N = 107	Class 2<br>N = 113	Class 3<br>N = 235	Class 4<br>N = 179
SEX				
Female	75 (70%)	69 (61%)	167 (71%)	117 (65%)
Male	24 (22%)	32 (28%)	48 (20%)	44 (25%)
Other/Unknown	<10	12 (11%)	20 (8.5%)	18 (10%)
RACE				
White	66 (62%)	58 (51%)	142 (60%)	103 (58%)
Hispanic/Latino	12 (11%)	15 (13%)	33 (14%)	32 (18%)
Multiracial	9 (8.4%)	7 (6.2%)	18 (7.7%)	10 (5.6%)
Black	<10	<10	<10	<10
Asian	<10	<10	<10	<10
Other/Unknown	10 (9.3%)	21 (19%)	30 (13%)	25 (14%)
ED or Hospital Visit for COVID-19				
No	65 (61%)	46 (41%)	124 (53%)	72 (40%)
Yes	42 (39%)	67 (59%)	111 (47%)	107 (60%)
AGE				
50 and Older	52 (49%)	77 (68%)	125 (53%)	107 (60%)
Under 50	55 (51%)	36 (32%)	110 (47%)	72 (40%)

Supplementary Table 1. Demographic characteristics by LCA Class assignment. Counts and percentages of participants assigned to each LCA class are shown for demographic characteristics Sex, Race, and Age, as well as ED or hospital visit for COVID-19.

Supplementary Figure 4.

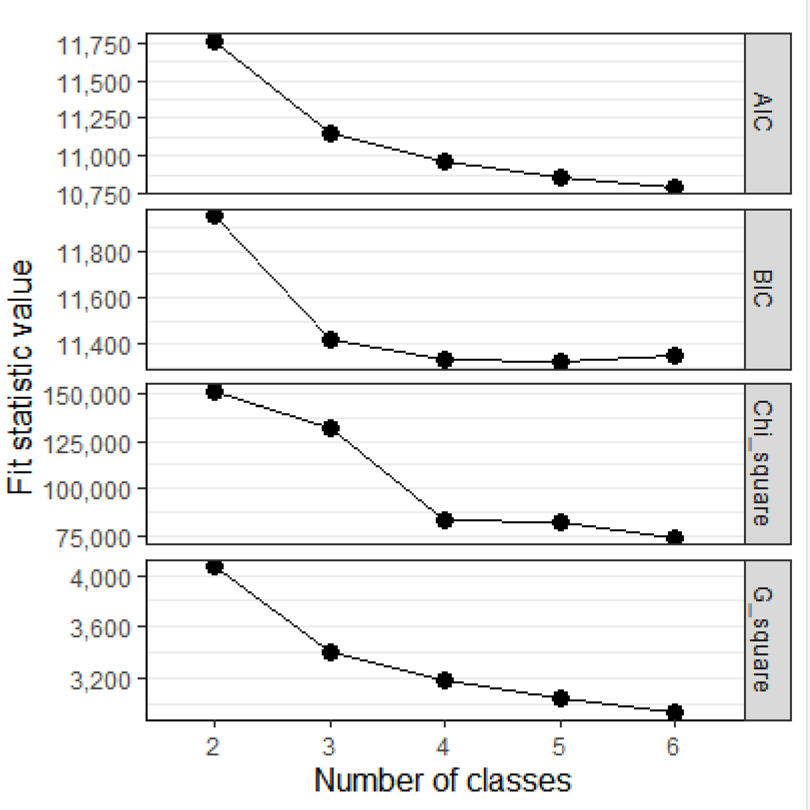

Supplementary Figure 4. Fit statistics for LCA models with number of classes varying from 2 to 6. Statistics included Akaike information criterion, (AIC), Bayesian information criterion (BIC), Pearson's Chi-square goodness of fit, and likelihood ratio chi-square (G_square). In this analysis, the AIC and G^2 statistics both decreased sequentially with the inclusion of each additional class, but the BIC reached a minimum at five classes, while the Chi-square statistic showed a significant drop from three to four classes. Moreover, neither the Chi-square nor the BIC statistic showed a significant decrease when advancing from four to five classes. Furthermore, models with more than four classes resulted in class sizes below 100, risking overfitting and unbalanced distribution of class sizes. This analysis suggested that the model with four classes was the optimal fit to the data for the purposes of this study. Classification accuracy was also evaluated using two classification diagnostics. The optimal model had a relative entropy of 0.85 and average posterior class probabilities (APP) above 0.87 for each class (values closer to 1 for both diagnostics indicate higher accuracy)[1].

Supplementary Table 2. Population shares and fit statistics for the optimal four-class LCA model.

	Class 1	Class 2	Class 3	Class 4
Estimated class population shares	0.174	0.285	0.195	0.346
Predicted class population shares	0.175	0.284	0.199	0.342

Model Statistics	
Number of observations	634
Number of estimated parameters	83
Residual degrees of freedom	551
Maximum log-likelihood	-5398.67
IC(4)	10963.34
BIC(4)	11332.86
G^2(4) (Likelihood ratio/deviance statistic)	3179.286
X^2(4) (Chi-square goodness of fit)	77714.35

Supplementary Table 2. Population shares and fit statistics for the optimal four-class LCA model. Estimated and predicted population shares are shown in the top rows of the table. Estimated shares are computed by the LCA model, predicted shares are computed after class assignment based on posterior probabilities. Model fit statistics include counts of observations, parameters, degrees of freedom, and optimal fit statistics.

Supplementary Table 3. Average posterior class probabilities (APP) for the optimal model with 4 classes.

	Probability of being assigned to class X			
Latent Class Assigned	1	2	3	4
1	94.3%	2.3%	3.4%	0.0%
2	1.1%	87.0%	8.9%	2.9%
3	1.0%	7.0%	89.9%	2.2%
4	0.0%	1.3%	2.1%	96.6%

Supplementary Table 3. Average posterior class probabilities (APP) for the optimal model with 4 classes. Higher diagonal values (i.e., closer to 1.0) and lower values off the diagonal indicate better accuracy. Some researchers use a 0.80 cutoff for acceptable diagonal probabilities. Others suggest a cutoff value of greater than 0.90[2].

Supplementary Table 4. Conditional Class Probabilities determined by the four-class LCA model, by PROMIS-10 domain.

	Low	Medium	High
General Health			
Class 1	0.0%	17.6%	82.4%
Class 2	47.5%	48.4%	4.1%
Class 3	7.5%	89.4%	3.1%
Class 4	94.3%	5.7%	0.0%
Quality Of Life			
Class 1	0.0%	11.7%	88.3%
Class 2	20.0%	65.2%	14.8%
Class 3	11.5%	82.5%	6.0%
Class 4	81.5%	17.8%	0.7%
Physical Health			
Class 1	3.0%	33.0%	63.9%
Class 2	75.7%	24.3%	0.0%
Class 3	29.0%	68.7%	2.3%
Class 4	98.8%	1.2%	0.0%
Mental Health			
Class 1	23.3%	37.5%	39.2%
Class 2	6.0%	54.8%	39.2%
Class 3	59.5%	38.8%	1.6%
Class 4	84.6%	14.6%	0.8%
Social Satisfaction			
Class 1	13.7%	31.3%	55.0%
Class 2	16.4%	62.0%	21.7%
Class 3	53.2%	45.2%	1.6%
Class 4	88.5%	11.5%	0.0%
Usual Social Activities			
Class 1	10.6%	24.2%	65.2%
Class 2	32.8%	52.1%	15.1%
Class 3	42.1%	50.1%	7.8%
Class 4	92.3%	7.7%	0.0%
Everyday Physical Activities			
Class 1	8.8%	6.4%	84.8%
Class 2	29.8%	47.2%	23.0%
Class 3	7.0%	37.1%	56.0%
Class 4	53.1%	34.8%	12.1%
Emotional Problems			
Class 1	49.6%	16.2%	34.2%
Class 2	2.8%	38.8%	58.4%
Class 3	47.5%	44.3%	8.2%
Class 4	56.9%	37.7%	5.4%
Fatigue			
Class 1	11.5%	33.0%	55.5%
Class 2	16.1%	55.3%	28.6%
Class 3	23.4%	50.1%	26.5%
Class 4	45.3%	50.6%	4.0%
Pain			
Class 1	26.8%	6.3%	66.9%
Class 2	23.9%	31.4%	44.7%
Class 3	12.9%	19.2%	67.8%
Class 4	30.8%	33.0%	36.2%

Supplementary Table 4. Conditional Class Probabilities determined by the four-class LCA model, by PROMIS-10 domain. Percentages in each row represent the probability of reporting Low, Medium, or High T scores given membership in an LCA class.

Supplementary Figure 5.

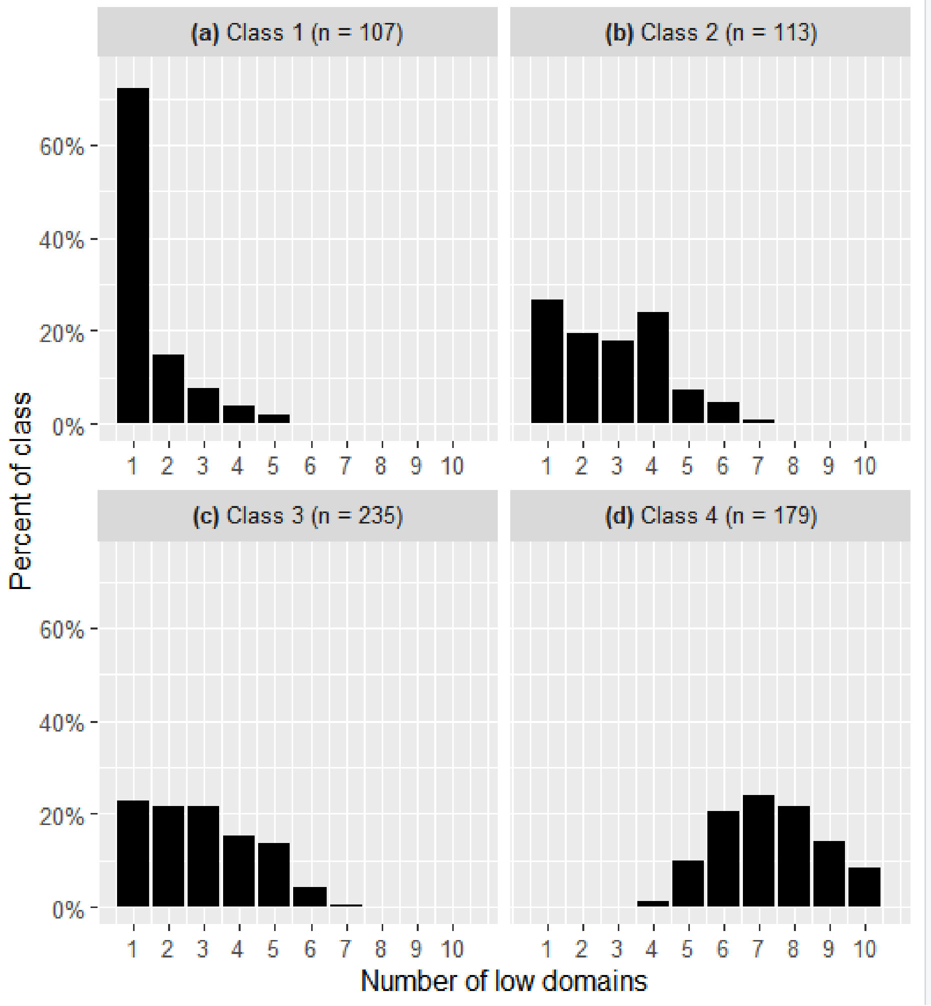

Supplementary Figure 5. Number of participants reporting ‘Low’ domains, as percent of LCA class, for 1 to 10 PROMIS-10 domains.

Supplementary Figure 6.

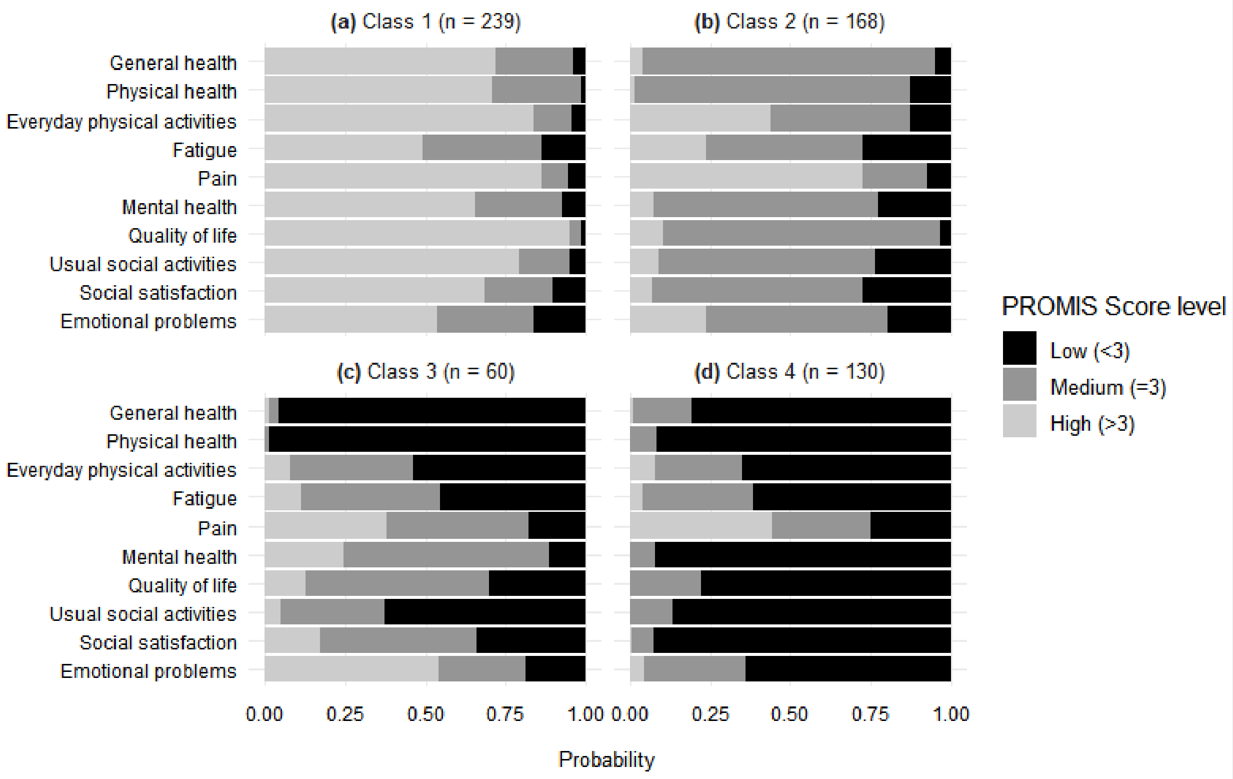

Supplementary Figure 6. LCA probabilities for PROMIS-10 Scores for study participants at 1-4 Weeks (N=597). Study participant responses at 1-2 weeks were analyzed by LCA. Patterns of PROMIS-10 component responses at 1-4 weeks did not mirror the patterns found for participants at 6 months.

Supplementary Methods 1.

1. In general, would you say your health is:

- Poor
- Fair
- Good
- Very good
- Excellent

2. In general, would you say your quality of life is:

- Poor
- Fair
- Good
- Very good
- Excellent

3. In general, how would you rate your physicial health?

- Poor
- Fair
- Good
- Very good
- Excellent

4. In general, how would you rate your mental health, including your mood and your ability to think?

- Poor
- Fair
- Good
- Very good
- Excellent

5. In general, how would you rate your satisfaction with your social activities and relationships?

- Poor
- Fair
- Good
- Very good
- Excellent

6. In general, please rate how well you carry out your usual social activities and roles. (This includes activities at home, at work and in your community, and responsibilities as a parent, child, spouse, employee, friend, etc.)

- Poor
- Fair
- Good
- Very good
- Excellent

7. To what extent are you able to carry out your everyday physical activities such as walking, climbing stairs, carrying groceries, or moving a chair?

- Not at all
- A little
- Moderately
- Mostly
- Completely

8. In the past 7 days, how often have you been bothered by emotional problems such as feeling anxious, depressed or irritable?

- Always
- Often
- Sometimes
- Rarely
- Never

9. In the past 7 days, how would you rate your fatigue on average?

- Very Severe
- Severe
- Moderate
- Mild
- None

10. In the past 7 days, how would you rate your pain on average?

- 0 – No Pain
- 1
- 2
- 3
- 4
- 5
- 6
- 7
- 8
- 9
- 10 – Worst Pain Imaginable

SUPPLEMENTARY REFERENCES

1. Weden, M. M. & Zabin, L. S. Gender and Ethnic Differences in the Co-occurrence of Adolescent Risk Behaviors. *Ethn Health* **10**, 213–234 (2005).

2. Weller, B. E., Bowen, N. K. & Faubert, S. J. Latent Class Analysis: A Guide to Best Practice. *Journal of Black Psychology* **46**, 287–311 (2020).
